# Supplementary material for: Multi-ancestry meta-analysis of keloids uncovers novel susceptibility loci in diverse populations
Source: Nat Commun. 2025 Aug 20;16:7770. doi: 10.1038/s41467-025-62945-x (PMC12368108; doi:10.1038/s41467-025-62945-x)
Supplement: Supplementary file 1 — Supplementary Information [file 41467_2025_62945_MOESM1_ESM.pdf]

Supplementary Figures & Tables

Ancestry	Case definition	Population definition	*N* Cases	*N* Controls	*N* Total
African/African American	ICD-9 code 701.4, ICD-10 code L91.0	GIA* (AFR)	985	57,858	58,843
American Admixed/Latino	ICD-9 code 701.4, ICD-10 code L91.0	GIA (AMR)	387	52,812	53,199
East Asian	ICD-9 code 701.4, ICD-10 code L91.0	GIA (EAS)	117	6,637	6,754
European	ICD-9 code 701.4, ICD-10 code L91.0	GIA (EUR)	1,882	171,131	173,013
Total			3371	288,438	291,809

Supplementary Table 1. Sample sizes for each of the ancestry groups utilized in the All of Us replication. European=EUR; East Asian=EAS; African=AFR.

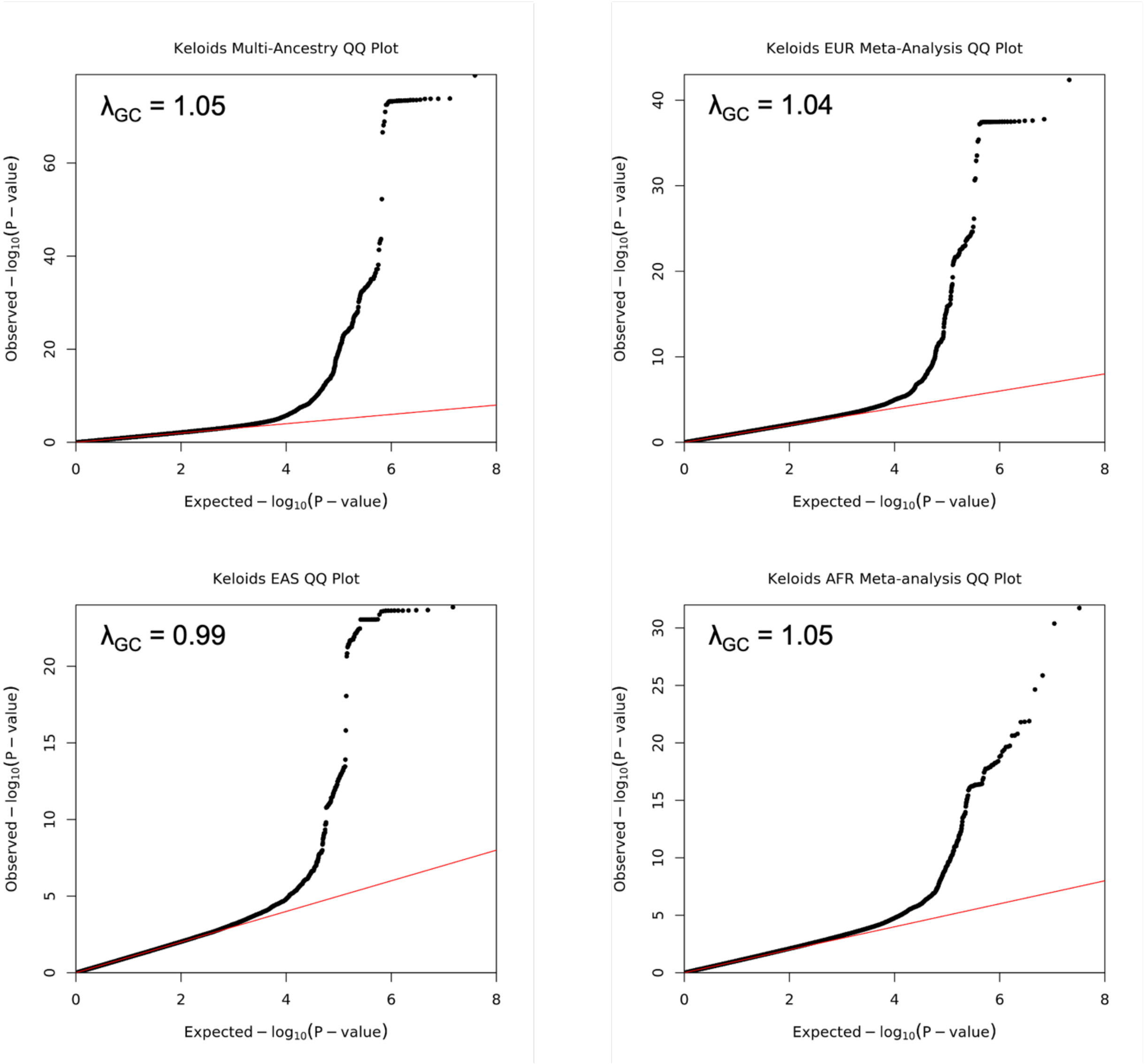

Supplementary Figure 1. Quantile-quantile (Q-Q) plots comparing the observed distribution of p-values from each meta-analysis against an expected normal distribution. Plots created (including λ_{GC} values) using fastman R library.

SNP	Multi-ancestry	EUR	AFR	EAS
rs10863683	$1.52×10^{-79}$	$1.32×10^{-44}$	$5.81×10^{-19}$	$1.45×10^{-21}$
rs11293015	$1.28×10^{-69}$	$6.56×10^{-36}$	$8.72×10^{-16}$	$1.39×10^{-24}$
rs1511412	0.0003951	0.09656	0.7626	$2.00×10^{-08}$
rs16976600	$1.41×10^{-26}$	$1.82×10^{-08}$	$5.16×10^{-11}$	$3.49×10^{-14}$
rs192314256	$8.74×10^{-19}$	NA	NA	$8.74×10^{-19}$
rs2378519	$1.68×10^{-74}$	$5.85×10^{-40}$	$3.65×10^{-17}$	$4.20×10^{-24}$
rs646315	$2.36×10^{-05}$	0.01969	0.1415	$4.63×10^{-10}$
rs74983791	$7.43×10^{-06}$	0.6763	0.001501	$6.36×10^{-07}$
rs8032158	$1.38×10^{-25}$	$3.80×10^{-09}$	$1.73×10^{-09}$	$3.39×10^{-13}$
rs873549	$3.76×10^{-74}$	$3.05×10^{-40}$	$5.33×10^{-17}$	$8.97×10^{-24}$

Categories

Category
Significant ($p<5×10^{-8}$)
Suggestive ($p<1×10^{-5}$)
Not significant
Not sampled

Supplementary Table 2. Replication status of previously identified genome-wide significant ($p<5×10^{-8}$) SNPs. Of the 10 previously identified variants, seven were replicated at genome-wide significance in the cross-ancestry analysis. The remaining three variants had suggestive evidence of association. Logistic regression statistical tests; multiple testing correction p-value threshold used (5 × 10−8).

							Frequency	
**Ancestry**	**SNP**	**Chr:BP**	**Effect**	**Ref**	**OR (95% CI)**	**P-value**	**GWAS**	**gnomAD**
EUR	rs35383942	1:201437832	T	C	1.49 (1.38 — 1.61)	1.11×10^{-23}	0.0862	0.1185
EUR	rs10863683	1:222251089	C	G	1.39 (1.33 — 1.46)	1.32×10^{-44}	0.3310	0.3236
EUR	rs4980386	11:1895708	C	A	1.15 (1.10 — 1.20)	1.12×10^{-09}	0.6081	0.6126
EUR	rs8032158	15:56194877	C	T	1.15 (1.10 — 1.20)	3.80×10^{-09}	0.3122	0.3180
EUR	rs34647667	15:68789866	T	G	1.30 (1.21 — 1.39)	6.18×10^{-14}	0.8569	0.8482
EUR	rs72905568	18:42245105	C	T	1.30 (1.19 — 1.41)	2.15×10^{-09}	0.9154	0.9190
EUR	rs56238684	20:33236696	C	G	1.33 (1.22 — 1.44)	7.87×10^{-11}	0.0733	0.0733
EAS	rs192314256	1:201437730	C	T	6.21 (4.14 — 9.30)	8.74×10^{-19}	0.0152	0.0133
EAS	rs10863683	1:222251089	C	G	1.61 (1.46 — 1.78)	1.45×10^{-21}	0.3187	0.3542
EAS	rs11293015	1:222267442	G	GT	0.60 (0.54 — 0.66)	1.39×10^{-24}	0.7133	0.6499
EAS	rs374569112	1:223403377	G	GC	2.42 (1.62 — 3.63)	1.85×10^{-05}	0.0136	0.0083
EAS	rs646315	3:138842704	T	G	1.75 (1.47 — 2.09)	4.63×10^{-10}	0.0707	0.0349
EAS	rs16976600	15:56154635	T	C	1.43 (1.30 — 1.56)	3.49×10^{-14}	0.3564	0.3058
AFR	rs10863683	1:222251089	C	G	1.34 (1.26 — 1.43)	5.81×10^{-19}	0.2753	0.2506
AFR	rs191467669	1:222501109	A	G	1.79 (1.50 — 2.14)	1.01×10^{-10}	0.0202	0.0113
AFR	rs76024540	11:2920108	T	C	1.47 (1.35 — 1.61)	2.54×10^{-18}	0.1085	0.1188
AFR	rs59382910	15:56273565	CT	C	1.27 (1.19 — 1.35)	1.13×10^{-12}	0.3372	0.3545
AFR	rs34647667	15:68789866	T	G	1.48 (1.39 — 1.58)	1.83×10^{-32}	0.6576	0.6481
AFR	rs140716753	20:11106003	A	C	1.72 (1.50 — 1.98)	2.35×10^{-14}	0.0333	0.0356
AFR	rs10854317	21:29817983	A	G	1.39 (1.25 — 1.55)	3.78×10^{-09}	0.8815	0.8825

Supplementary Table 3. Lead SNPs for each of the ancestry-specific analyses (European=EUR; East Asian=EAS; African=AFR). All alleles and effect sizes are reported in the risk-increasing direction, except for the EAS indel rs11293015. Logistic regression statistical tests; multiple testing correction p-value threshold used (5×10^{-8}).

Group	Genomic Inflation Factor lambda	LDSC Intercept (SE)	LDSC heritability (SE)
Multi-ancestry	1.05	1.03 (0.006)	0.06 (0.01)
EUR	1.04	1.03 (0.007)	0.06 (0.02)
EAS	0.99	0.98 (0.007)	0.19 (0.06)
AFR	1.05	1.03 (0.007)	0.34 (0.07)

Supplementary Table 4. Genomic inflation factor, LDSC intercept, and SNP-based heritability estimate. The Lambda GC was calculated using the non-filtered set of summary statistics during creation of the QQ plots with the R fastman package.

			Datasets represented		
Chr	BP	SNP	EUR	EAS	AFR
1	22702231	rs12568930	Y	Y	Y
1	201437832	rs35383942	Y	N	Y
1	222251089	rs10863683	Y	Y	Y
1	222291773	rs140707031	N	N	Y
2	28381833	rs6726716	Y	Y	Y
2	241252201	rs12989123	Y	Y	Y
3	138837253	rs75826502	N	Y	N
5	88095785	rs244755	Y	Y	Y
6	149664540	rs6906384	Y	Y	Y
7	37940286	rs2242026	Y	Y	Y
8	32555685	rs2919386	Y	Y	Y
8	126534536	rs921721	Y	Y	Y
9	4287190	rs6476838	Y	Y	Y
11	1891722	rs686722	Y	Y	Y
11	2920108	rs76024540	N	N	Y
12	106119041	rs7297246	Y	Y	Y
15	56210499	rs11632096	Y	Y	Y
15	68789866	rs34647667	Y	Y	Y
18	42241036	rs77685836	Y	N	Y
20	10670079	rs2423510	Y	Y	Y
20	11106003	rs140716753	N	N	Y
20	11242516	rs4239705	Y	Y	Y
20	32849416	rs1205312	Y	N	Y
20	49984404	rs6091310	Y	Y	Y
21	29816067	rs13051336	Y	Y	Y
21	30132507	rs2832056	Y	Y	Y
X	20810164	rs769545468	N	N	Y

Supplementary Table 5. Distribution of lead genome-wide significant SNPs across ancestry-specific analyses. Population-specific SNPs (ie, those represented at MAF>1% in only one ancestry-specific meta-analysis) are in bold.

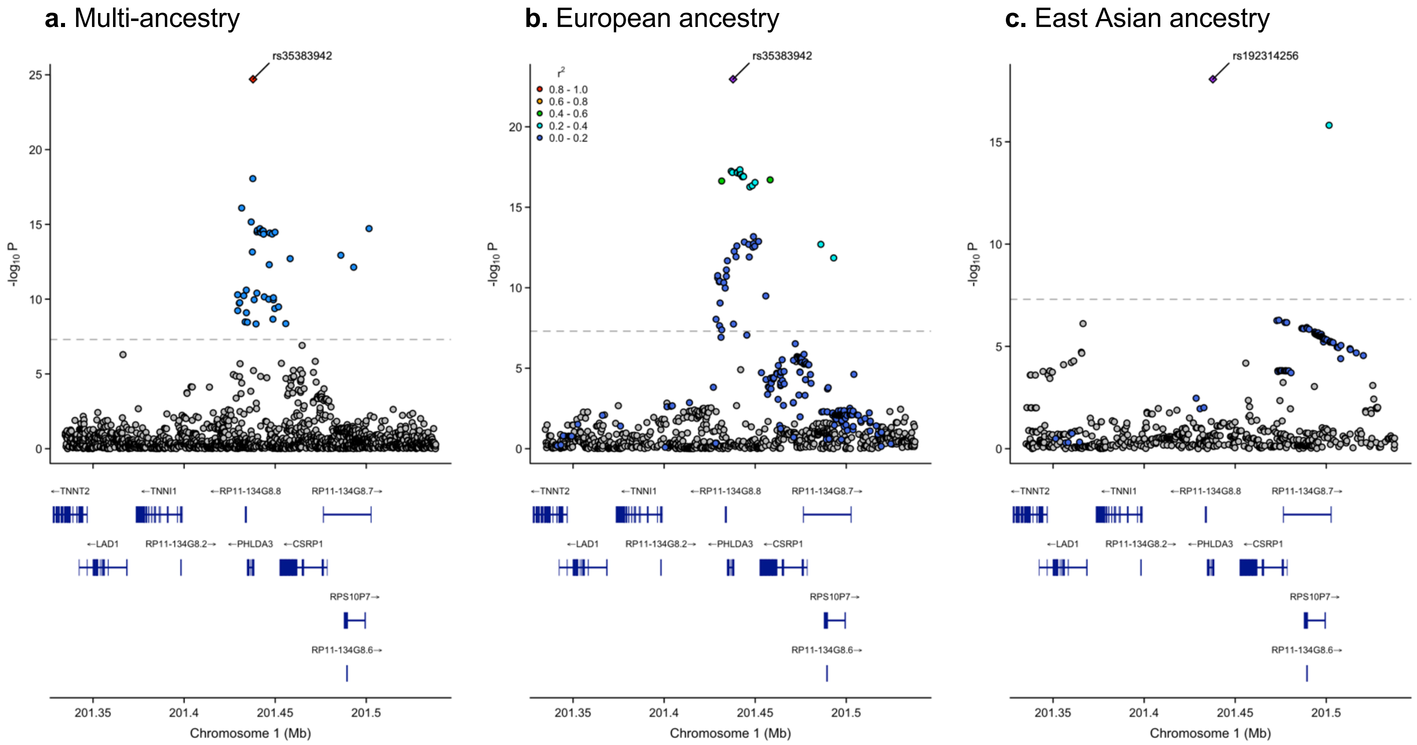

Supplementary Figure 2. Independent genome-wide significant SNPs mapping to *PHLDA3* on chr1. Ancestry-specific analyses (**b** and **c**) also display population-specific linkage disequilibrium information. The variant rs192314256 in the East Asian analysis is relatively rare, at just above 1% in the East Asian population; The variant rs35383942 in the multi-ancestry and European ancestry analyses, meanwhile, is vanishingly rare (<0.01%) in East Asian populations. Logistic regression statistical tests; multiple testing correction p-value threshold used (5×10^{-8}).

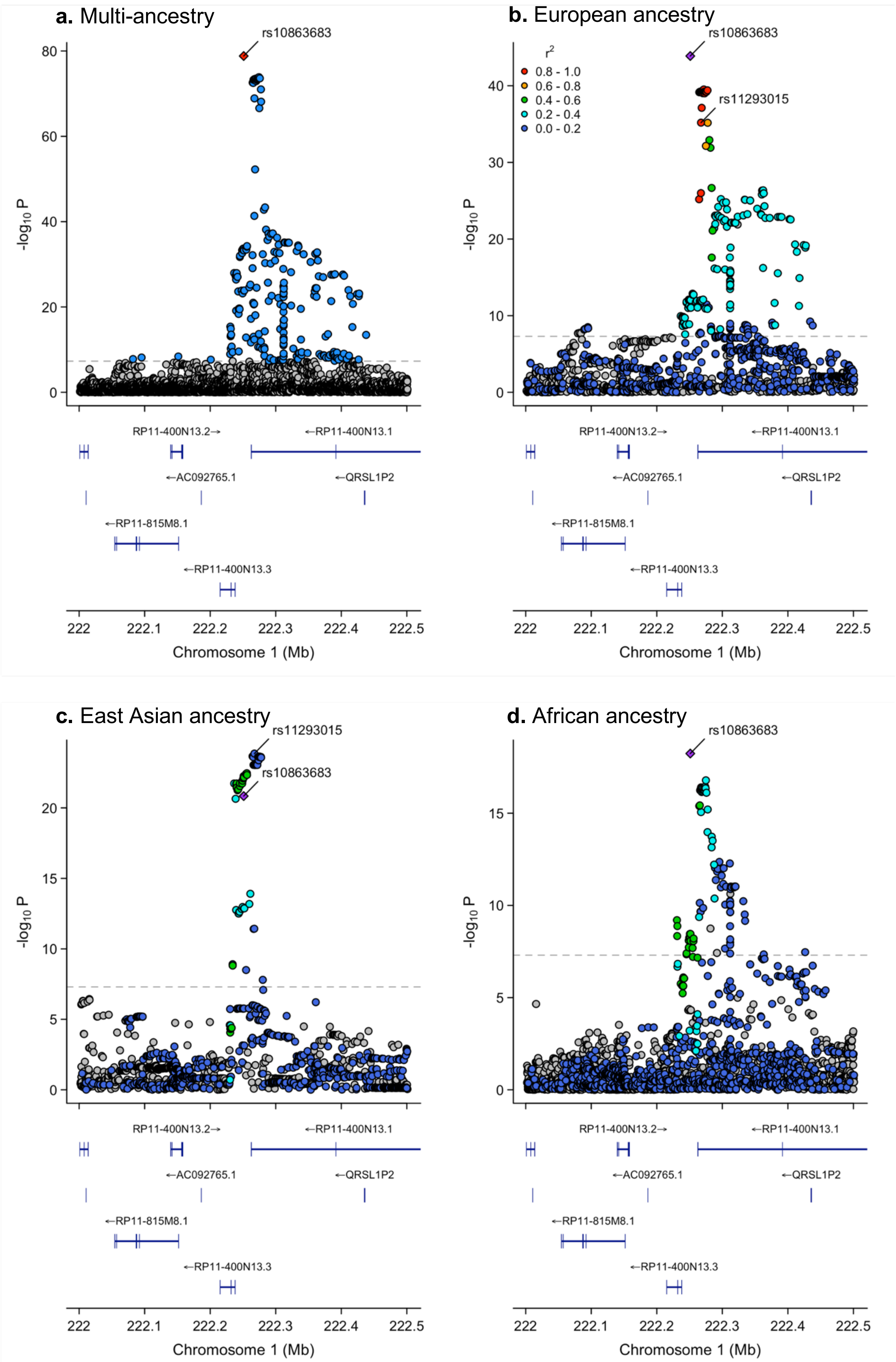

Supplementary Figure 3. Independent genome-wide significant SNPs mapping to *RP11-415K20.1 / LINC01705* on chr1. Across all analyses, rs10863683 is a conditionally independent lead SNP. **b.** Variant rs10863683 is in linkage disequilibrium with rs11293015 in European populations and does not constitute a separate genomic risk locus in Europeans. **c.** The most significant SNP in the East Asian analysis was rs11293015, shown to be in linkage equilibrium with rs10863683 in East Asian populations. **d.** The variant rs191467669 was conditionally independent from rs10863683 in the African ancestry analysis. However, it is not present in the 1000 Genomes LD reference and is not shown here. Logistic regression statistical tests; multiple testing correction p-value threshold used (5×10^{-8}).

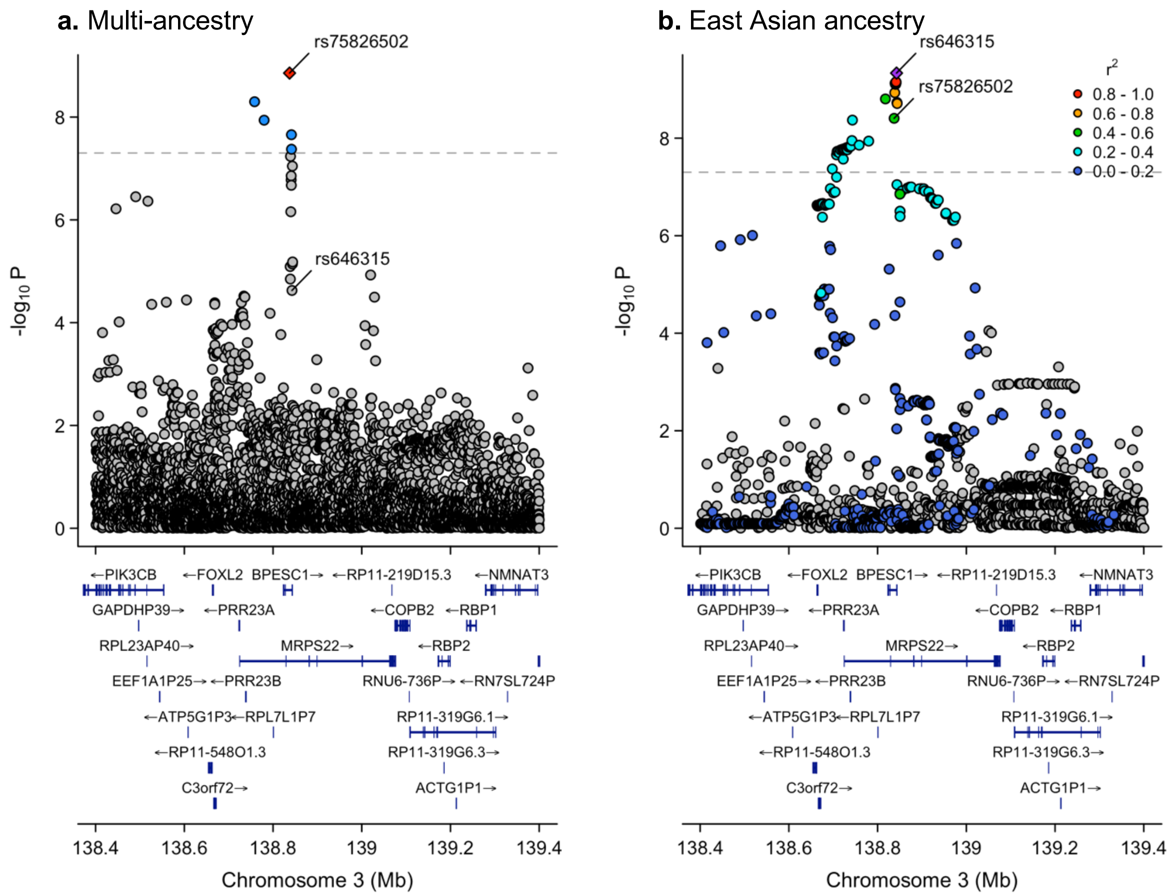

Supplementary Figure 4. Independent genome-wide significant SNPs mapping to *MRPS22 / BPESC1* on chr3. Variant rs75826502 is the index SNP for this locus in the multi-ancestry analysis (a.), as the most significant SNP in the East Asian analysis (rs646315, **b**) was not well-supported by other datasets. This disparity does not appear to be due to allele frequency limitations in non-Asian populations, however – East Asians have the lowest population frequency of the effect allele, T (~3%). Logistic regression statistical tests; multiple testing correction p-value threshold used (5×10^{-8}).

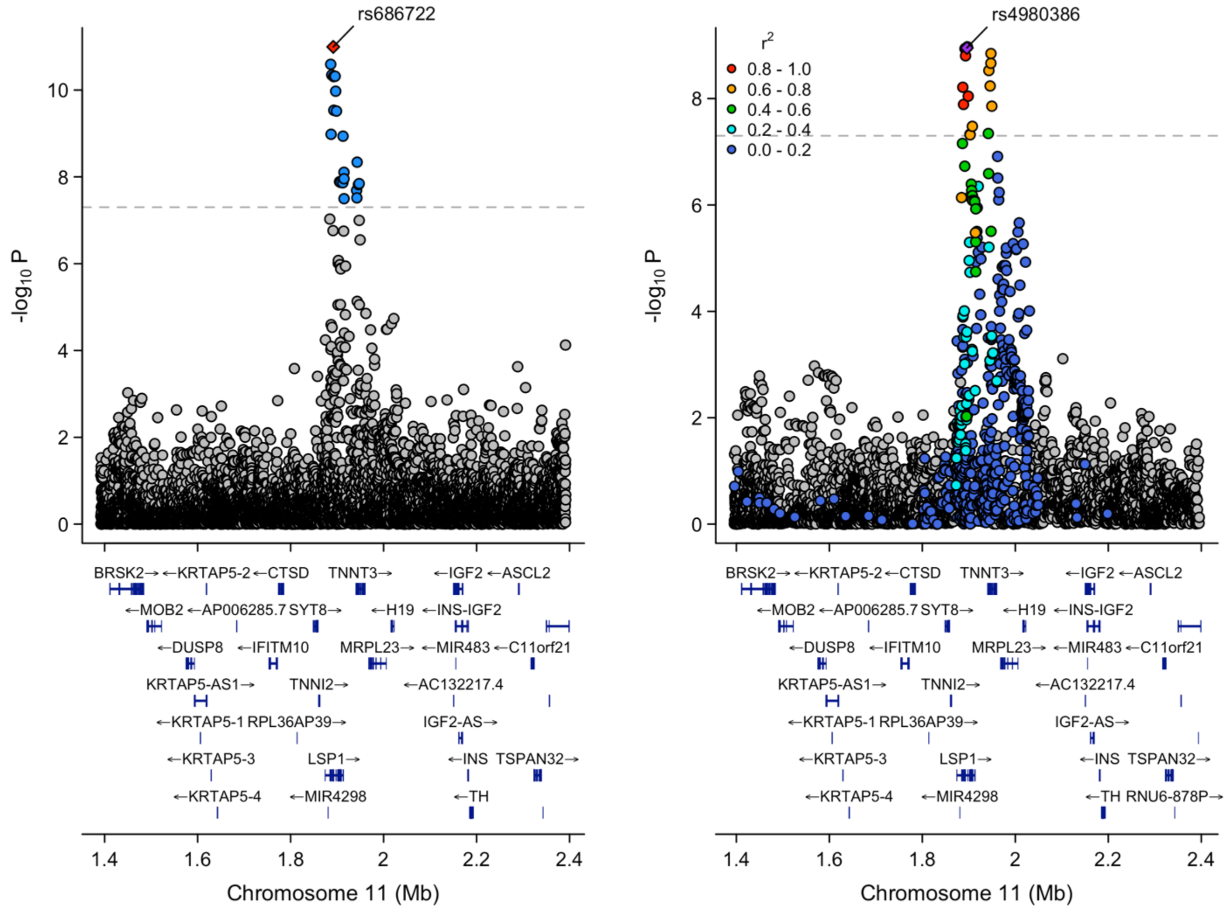

Supplementary Figure 5. Independent genome-wide significant SNPs mapping to *LSP1* on chr11. There are different lead SNPs for the multi-ancestry (**a**) versus European ancestry analyses (**b**), which are at similar positions (approximately 4 kb apart). Logistic regression statistical tests; multiple testing correction p-value threshold used (5×10^{-8}).

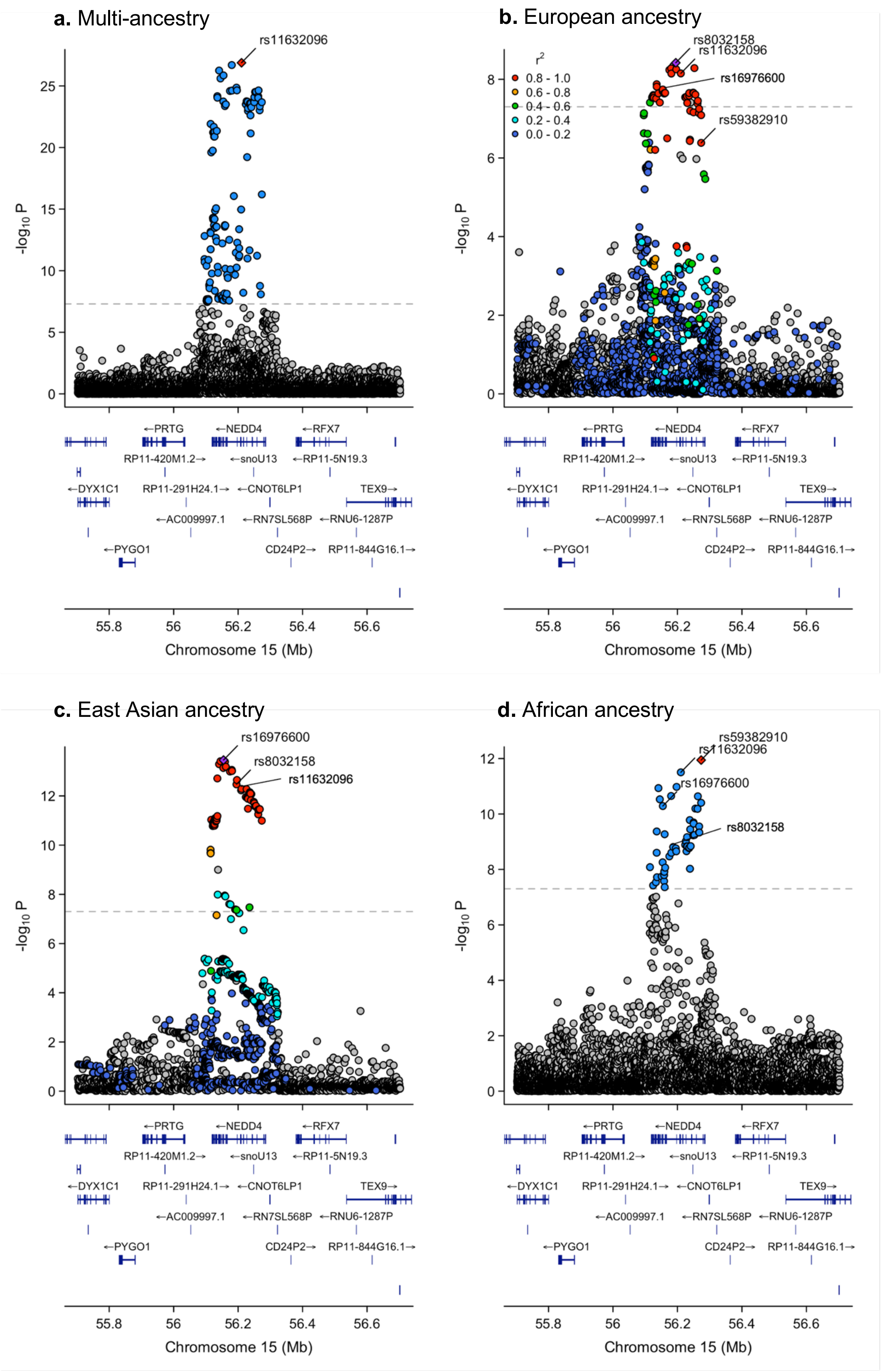

Supplementary Figure 6. Independent genome-wide significant SNPs mapping to *NEDD4* on chr15. Each analysis had a different lead SNP representing the genomic risk locus at *NEDD4*, though they are largely in high LD with each other. All lead SNPs for each of the ancestry-specific analyses (**b-d**) are labeled, if available. The exception is the African ancestry analysis, which has other SNPs in low LD with rs59382910. Logistic regression statistical tests; multiple testing correction p-value threshold used (5×10^{-8}).

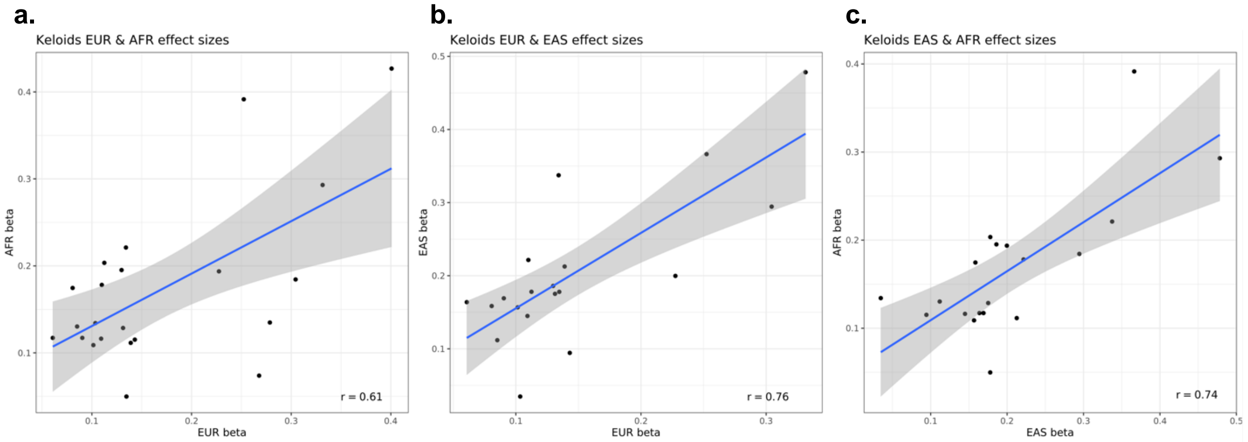

Supplementary Figure 7. Effect size comparisons between ancestry-specific analyses, restricted to SNPs in common between each pairwise analysis. Alleles were aligned to the same risk direction for each comparison. **a.** Comparing European and African effect sizes. **b.** Comparing European and East Asian effect sizes. **c.** Comparing East Asian and African effect sizes. r=Pearson correlation between SNP effect sizes; effect sizes represented as beta (β) values rather than as Odds Ratios, listed in Table 2. Line of best fit (blue line) and 95% confidence bands (shaded area) are shown for each comparison. Plot created using ggplot2 in R.

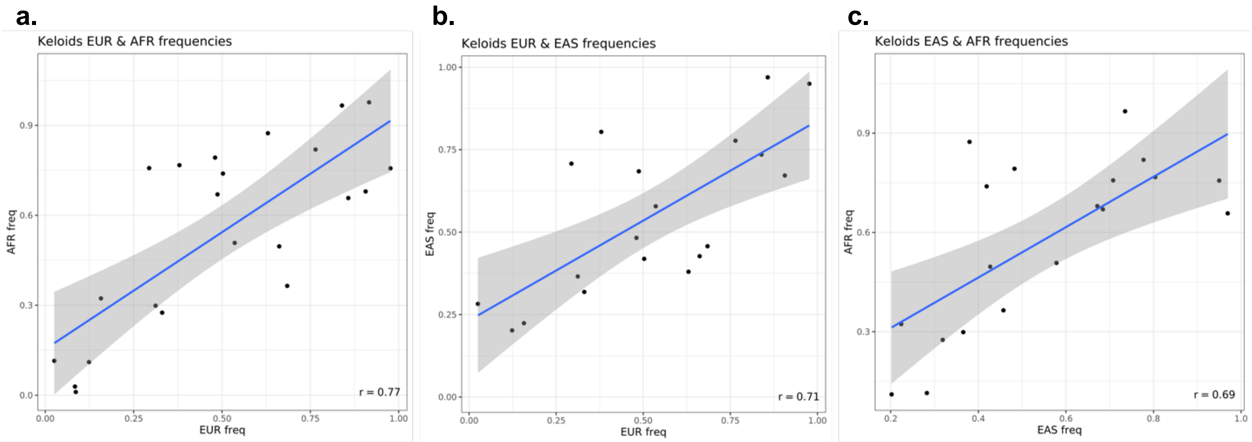

Supplementary Figure 8. Allele frequency comparisons between ancestry-specific analyses, restricted to SNPs in common between each pair. Alleles were aligned to the same risk direction for each comparison. **a.** Comparing European and African allele frequencies. **b.** Comparing European and East Asian allele frequencies. **c.** Comparing East Asian and African allele frequencies. r=Pearson correlation between SNP allele frequencies; freq=allele frequency; frequency reported is the population-specific allele frequency of the risk-increasing allele. Line of best fit (blue line) and 95% confidence bands (shaded area) are shown for each comparison. Plot created using ggplot2 in R.
