# Supplementary material for: Multi-ancestry meta-analysis of keloids uncovers novel susceptibility loci in diverse populations
Source: Nat Commun. 2025 Aug 20;16:7770. doi: 10.1038/s41467-025-62945-x (PMC12368108; doi:10.1038/s41467-025-62945-x)
Supplement: Supplementary file 2 — Description of Additional Supplementary Files [file 41467_2025_62945_MOESM2_ESM.pdf]

Description of Additional Supplementary Files

Supplementary Data 1

- **a.** Significant independent SNPs associated with keloid scars in the multi-ancestry analysis. Logistic regression statistical tests; multiple testing correction p-value threshold used (5×10^{-8}).
- **b.** Significant independent SNPs associated with keloid scars in the European-ancestry analysis. Logistic regression statistical tests; multiple testing correction p-value threshold used (5×10^{-8}).
- **c.** Significant independent SNPs associated with keloid scars in the East Asian-ancestry analysis. Logistic regression statistical tests; multiple testing correction p-value threshold used (5×10^{-8}).
- **d.** Significant independent SNPs associated with keloid scars in the African-ancestry analysis. Logistic regression statistical tests; multiple testing correction p-value threshold used (5×10^{-8}).

Supplementary Data 2 – Results for each of the 39 lead SNPs identified across the various meta-analyses. Significant ($p<0.05/39$) results are highlighted in green, suggestive ($p<0.05$) results are highlighted in orange. Variants not represented in All of Us or for which no proxy could be found (for any group) are in red text. EUR=European, EAS=East Asian, AFR=African, AMR=Admixed American. Logistic regression statistical tests; multiple testing correction p-value threshold used (5×10^{-8}).

Supplementary Data 3 – Cross-population fine-mapping, regions defined using unique lead SNPs from multi-ancestry and ancestry-specific analyses. Unique SNPs with PIP>0.9 are noted.

Supplementary Data 4 – FUMA SNP2GENE results for nonsynonymous variants in LD ($r2>0.1$) with FUMA lead SNPs. LD estimates (from FUMA and from LDlink) are shown, as are gnomAD population frequency estimates. Rreported effect alleles are those that increase keloids risk. Amino acid change obtained from the Ensembl Variant Effect Predictor (VEP). 'NA' values indicate that alleles do not occur in the given population, or that the allele was not in the reference.

Supplementary Data 5 – FUMA SNP2GENE results (multi-ancestry analysis) for Combined Annotation Dependent Depletion (CADD); RegulomeDB (RDB); and Chromatine 15 interaction values (E126, adult dermal fibroblasts).

Supplementary Data 6

- **a.** FUMA Significant Tissue Specificity, using input genes from multi-ancestry GWAS. Differentially expressed gene sets are from GTEx v8 53 tissue types. Hypergeometric statistical tests; adjusted p-value with threshold (0.05).
- **b.** FUMA Significant Gene Ontology Biological Pathway enrichment of significant variants of the multi-ancestry analysis. Hypergeometric statistical tests; adjusted p-value with threshold (0.05).
- **c.** FUMA Significant Gene Ontology Biological Pathway enrichment of significant variants of the EUR analysis
- **d.** FUMA Significant Gene Ontology Biological Pathway enrichment of significant variants of the EAS analysis. Hypergeometric statistical tests; adjusted p-value with threshold (0.05).

e. FUMA Significant Gene Ontology Biological Pathway enrichment of significant variants of the AFR analysis. Hypergeometric statistical tests; adjusted p-value with threshold (0.05).
f. FUMA Significant Gene Set Enrichment, using input genes from GWAS and GPGE. Hypergeometric statistical tests; adjusted p-value with threshold (0.05).

Supplementary Data 7 - All significant results across GPGE analyses, 49 tissues. Two-sided Wald test; multiple testing correction significance threshold $p<1.8\times10^{-7}$.

Supplementary Data 8

a. Combined GPGE results with p-value <0.05 and colocalization analysis results for multi-ancestry analysis.
b. Combined GPGE results with p-value <0.05 and colocalization analysis results for EUR analysis.
c. Combined GPGE results with p-value <0.05 and colocalization analysis results for EAS analysis.
d. Combined GPGE results with p-value <0.05 and colocalization analysis results for AFR analysis

Supplementary Data 9

a. IPA results of significant network enrichment, multi-ancestry analysis. Hypergeometric distribution testing with right-tailed Fisher's Exact Test; p-value threshold used (0.05).
b. IPA analysis of significant pathway enrichment, multi-ancestry analysis. Hypergeometric distribution testing with right-tailed Fisher's Exact Test; p-value threshold used (0.05).
c. IPA analysis of upstream regulators of data provided, multi-ancestry analysis. Hypergeometric distribution testing with right-tailed Fisher's Exact Test; p-value threshold used (0.05).
d. Top hits for each of the multi-ancestry and ancestry-specific IPA analyses. Hypergeometric distribution testing with right-tailed Fisher's Exact Test; p-value threshold used (0.05).

Supplementary Data 10 – Representation of significant GPGE genes in previously published RNA-seq datasets, obtained via a PubMed search for 'keloid' and 'rna-seq'. 18/32 studies had searchable supplementary materials.
