# Supplementary material for: Multi-ancestry meta-analysis of keloids uncovers novel susceptibility loci in diverse populations
Source: Nat Commun. 2025 Aug 20;16:7770. doi: 10.1038/s41467-025-62945-x (PMC12368108; doi:10.1038/s41467-025-62945-x)
Supplement: Supplementary file 13 — Reporting Summary [file 41467_2025_62945_MOESM13_ESM.pdf]

nature portfolio

Corresponding author(s): Jacklyn N. Hellwege

Last updated by author(s): July 28, 2025

Reporting Summary

Nature Portfolio wishes to improve the reproducibility of the work that we publish. This form provides structure for consistency and transparency in reporting. For further information on Nature Portfolio policies, see our Editorial Policies and the Editorial Policy Checklist.

Statistics

For all statistical analyses, confirm that the following items are present in the figure legend, table legend, main text, or Methods section.

n/a	Confirmed	
☐	☒	The exact sample size (n) for each experimental group/condition, given as a discrete number and unit of measurement
☒	☐	A statement on whether measurements were taken from distinct samples or whether the same sample was measured repeatedly
☐	☒	The statistical test(s) used AND whether they are one- or two-sided<br>*Only common tests should be described solely by name; describe more complex techniques in the Methods section.*
☐	☒	A description of all covariates tested
☐	☒	A description of any assumptions or corrections, such as tests of normality and adjustment for multiple comparisons
☐	☒	A full description of the statistical parameters including central tendency (e.g. means) or other basic estimates (e.g. regression coefficient) AND variation (e.g. standard deviation) or associated estimates of uncertainty (e.g. confidence intervals)
☐	☒	For null hypothesis testing, the test statistic (e.g. F, t, r) with confidence intervals, effect sizes, degrees of freedom and P value noted<br>*Give P values as exact values whenever suitable.*
☐	☒	For Bayesian analysis, information on the choice of priors and Markov chain Monte Carlo settings
☒	☐	For hierarchical and complex designs, identification of the appropriate level for tests and full reporting of outcomes
☐	☒	Estimates of effect sizes (e.g. Cohen's d, Pearson's r), indicating how they were calculated

Our web collection on statistics for biologists contains articles on many of the points above.

Software and code

Policy information about availability of computer code

Data collection	NA, all data were collected and de-identified prior to use.
Data analysis	All data analysis methods utilize publicly available and published software packages. The GWAS analyses of BioVU and eMERGE were completed using plink2. The analysis software to perform the meta-analyses was METAL v(2011-3-25). The SNP-based heritability and lambda intercept were estimated using LD Score Regression v1.0.1. Conditional analyses were conducted using Genome-wide Complex Trait Analysis v1.93.0. Functional annotations of enrichment tests of GWAS results were completed using Functional Mapping and Annotation Web tool (accessed November 2024). Predicted gene expression was completed using S-PrediXcan/MetaXcan v0.7.1. Gene expression colocalization was conducted using coloc R library v5.2.2. Pathway analysis of colocalized genes was completed with Ingenuity Pathway Analysis (accessed March 2024).<br><br>Follow-up analyses were conducted as follows: Fine-mapping analyses were performed using the Cross-population Sum of Single Effect Model (SuSiEx) in April 2025. The replication GWAS was conducted on the All of Us Researcher Workbench using the same methods described above for BioVU and eMERGE. Other additions consisted of edits to the text or to formatting of supplementary materials.

For manuscripts utilizing custom algorithms or software that are central to the research but not yet described in published literature, software must be made available to editors and reviewers. We strongly encourage code deposition in a community repository (e.g. GitHub). See the Nature Portfolio guidelines for submitting code & software for further information.

Data

Policy information about availability of data

All manuscripts must include a data availability statement. This statement should provide the following information, where applicable:

- Accession codes, unique identifiers, or web links for publicly available datasets
- A description of any restrictions on data availability
- For clinical datasets or third party data, please ensure that the statement adheres to our policy

The anonymised summary statistics will be available as open data and upon request. GWAS Catalog accession numbers: GCST90652487 (multi-ancestry), GCST90652488 (European), GCST90652489 (African). East Asian summary statistics are already publicly available via Biobank Japan, listed in the Data Availability section.

Research involving human participants, their data, or biological material

Policy information about studies with human participants or human data. See also policy information about sex, gender (identity/presentation), and sexual orientation and race, ethnicity and racism.

Reporting on sex and gender	Keloids affect both men and women, so no gender-based restriction was applied. Each contributing GWAS, including those performed by the authors in BioVU and eMERGE, included biological sex as a covariate in analyses.
Reporting on race, ethnicity, or other socially relevant groupings	Our data stratification included use of EHR- and self-reported race and ethnicity. We recognize that race and ethnicity are not ideal substitutes for genetic ancestry. We detail in table 1 whether each data source used race and ethnicity or genetic ancestry to determine the meta-analysis strata grouping. Non-Hispanic Black individuals were part of the African ancestry analyses, Non-Hispanic White and Finnish individuals were part of the European ancestry analyses, and Japanese individuals were part of the East Asian analyses.
Population characteristics	Case and control definitions for each data source varied, consisting of ICD billing codes, phecodes, and clinical notes. These are described in Table 1 and in the methods portion of the paper.
Recruitment	Each data source had its own recruitment method, all previously published. There are both hospital- and community-based recruitment methods utilized across the data sources. There may be some ascertainment bias due to these varying recruitment methods, and we have therefore prioritized reporting of results with evidence across multiple datasets.
Ethics oversight	This research was approved by the Vanderbilt University Medical Center Institutional Review Board.

Note that full information on the approval of the study protocol must also be provided in the manuscript.

Field-specific reporting

Please select the one below that is the best fit for your research. If you are not sure, read the appropriate sections before making your selection.

[x] Life sciences [ ] Behavioural & social sciences [ ] Ecological, evolutionary & environmental sciences

For a reference copy of the document with all sections, see nature.com/documents/nr-reporting-summary-flat.pdf

Life sciences study design

All studies must disclose on these points even when the disclosure is negative.

Sample size	We report the complete sample size for each data source and combined meta-analyses.
Data exclusions	Genetic quality control excluded low quality samples and was performed separately by each data source used. Samples were also excluded if consent had been revoked, sample was duplicated, or sex concordance checks failed.
Replication	Our study was able to replicate other previously reported keloid-associated variants and genes. Replication status for these previous findings are detailed in Supplementary Table 3. We also conducted replication analyses in an independent dataset, as requested by the reviewers. Details of this replication analysis can be found in the main text and in Supplementary Table 6.
Randomization	This is an observational study of associations between genetic variants and keloid scars. Randomization is not required, as case and control definitions are based on presence or absence of keloids.
Blinding	Blinding is not relevant to genome-wide association studies or to the subsequent statistical analyses performed in this study.

Reporting for specific materials, systems and methods

We require information from authors about some types of materials, experimental systems and methods used in many studies. Here, indicate whether each material, system or method listed is relevant to your study. If you are not sure if a list item applies to your research, read the appropriate section before selecting a response.

Materials & experimental systems

n/a	Involved in the study
☒	☐ Antibodies
☒	☐ Eukaryotic cell lines
☒	☐ Palaeontology and archaeology
☒	☐ Animals and other organisms
☒	☐ Clinical data
☒	☐ Dual use research of concern
☒	☐ Plants

Methods

n/a	Involved in the study
☒	☐ ChIP-seq
☒	☐ Flow cytometry
☒	☐ MRI-based neuroimaging

Plants

Seed stocks	*Report on the source of all seed stocks or other plant material used. If applicable, state the seed stock centre and catalogue number. If plant specimens were collected from the field, describe the collection location, date and sampling procedures.*
Novel plant genotypes	*Describe the methods by which all novel plant genotypes were produced. This includes those generated by transgenic approaches, gene editing, chemical/radiation-based mutagenesis and hybridization. For transgenic lines, describe the transformation method, the number of independent lines analyzed and the generation upon which experiments were performed. For gene-edited lines, describe the editor used, the endogenous sequence targeted for editing, the targeting guide RNA sequence (if applicable) and how the editor was applied.*
Authentication	*Describe any authentication procedures for each seed stock used or novel genotype generated. Describe any experiments used to assess the effect of a mutation and, where applicable, how potential secondary effects (e.g. second site T-DNA insertions, mosiacism, off-target gene editing) were examined.*
